# First Contact With Care Through Clinical Simulation in Nursing Students: Qualitative Study

**DOI:** 10.2196/81617

**Published:** 2025-10-08

**Authors:** Eva García Carpintero-Blas, Pablo Del Pozo-Herce, Maria del Carmen Hernández-Cediel, Marta Rodríguez-García, Noelia Navas-Echazarreta, Elena Chover-Sierra, Antonio Martínez-Sabater, Raúl Juárez-Vela, Alberto Tovar-Reinoso

**Affiliations:** 1 Research Group on Innovation in Health Care and Nursing Education (INcUidE), University of UNIE, C. de Arapiles, 14, Chamberí, Madrid, 28015, Spain, 34 919032201; 2 Department of Nursing, Faculty of Health Sciences, Research Group in Care GRUPAC, University of La Rioja, Logroño, Spain; 3 Nursing Care and Education Research Group (GRIECE), GIUV2019-456, Nursing Department, Universitat de Valencia, Valencia, Spain; 4 Internal Medicine, Consorci Hospital General Universitari de Valencia, Valencia, Spain; 5 Care Research Group (INCLIVA), Hospital Clínico Universitario de Valencia, Valencia, Spain

**Keywords:** clinical simulation, professional identity, experiential learning, standardized patients, nursing education

## Abstract

**Background:**

Clinical simulation with standardized patients provides nursing students with their first approach to care in a safe and realistic environment. This type of experience arouses intense emotions and supports the development of key competencies.

**Objective:**

This study aimed to explore the perceptions of nursing students during their first contact with care through simulation experiences with standardized patients.

**Methods:**

We conducted a qualitative descriptive phenomenological study using focus groups and reflective narratives with a sample of 59 students. A thematic analysis was performed using ATLAS.ti (version 24; Scientific Software Development GmbH).

**Results:**

Three thematic blocks, along with their categories and subcategories, were identified: (T1) first contact with simulation, (T2) learning and competencies, and (T3) preparation for clinical practice.

**Conclusions:**

Clinical simulation has a profound impact on nursing education by offering a safe and realistic environment in which to learn from experience. Emotions, teamwork, and human interaction enrich learning and strengthen professional identity. This approach supports the integration of technical knowledge and relational skills. The results support the inclusion of active and humanized methodologies in training plans.

## Introduction

### Background

The education of future nursing professionals requires a progressive, safe, and meaningful approach to the clinical environment. During the first years of the degree, students face the challenge of transforming theoretical knowledge into practical skills that enable them to provide effective and humanized care [1,2]. This transition process, especially in its initial stages, may generate uncertainty, anxiety, and doubts regarding their own competence and professional role [3]. Before their first entry into real clinical scenarios, students must have a first approach to care within a safe environment, which allows them to become familiar with basic procedures and the relationship with the patient [4,5]. In this context, the first contact with basic care represents a formative milestone of great relevance. Activities such as grooming, mobilization, taking vital signs, or feeding a dependent person, although they may seem simple from a technical point of view, involve a high emotional and relational load for the novice student. In turn, these first experiences can awaken insecurities, fears of error or judgment, especially when they have not been previously addressed in a pedagogical context of accompaniment and reflection [6].

Traditional approaches to teaching and learning in nursing have proven to be limited in effectively developing the necessary clinical competencies [7]. Therefore, there is a need to incorporate innovative training strategies that allow students to acquire realistic experiences in a safe environment, free of risk to patients [8]. In this sense, clinical simulation has gained relevance as a resource of high educational value, which can significantly improve cognitive learning, psychomotor skills, and emotional development of students, depending on the area and type of implementation [9-12]. Evidence further indicates that simulation-based education produces better learning outcomes than traditional educational methods [13]. Moreover, students who participate in multiple simulation sessions tend to develop a higher level of competence and self-confidence [14].

Simulation-based experience (SBE) can take multiple forms, from structured or virtual scenarios to hybrid modalities, classified by their level of realism [15]. Among these, simulation with standardized patients (SPs; individuals trained to realistically portray specific clinical conditions) offers a highly realistic environment [16]. In this context, it is important to distinguish between the term SP and the actor. According to authors such as Lioce et al [17], an “actor” refers to a person who performs a role in a simulation scenario, who may or may not be specifically trained to standardize a clinical condition. Thus, while a SP combines acting skills with knowledge of the assigned clinical case, the term actor is used in a more general sense.

Numerous studies have shown that the use of SPs contributes to the development of psychomotor skills, improves self-confidence, enhances critical thinking, and strengthens students’ communication and interpersonal skills [18,19]. Likewise, by incorporating emotional, ethical, and relational elements, it supports the construction of meaningful and humanized learning [20,21], especially in areas such as basic care, often underestimated in the hierarchy of clinical knowledge, but fundamental in the comprehensive care of the person.

Despite these benefits, most research has focused on advanced students or on high-fidelity contexts associated with critical situations [22]. There is scant evidence on how novice students, in their first year, experience and perceive this first contact with care through clinical simulation [1]. Understanding these initial experiences from the student’s own voice is essential to adjust pedagogical strategies, promote adequate emotional support, and facilitate a more conscious, safe, and empathetic transition to real clinical practice [23].

From a qualitative and phenomenological approach, this study aims to understand the perceptions, emotions, and learning of first-year nursing students during their first clinical simulation experience with SPs, focused on basic care. The aim is to give visibility to the subjective and formative impact of this type of experience, as well as to identify elements that favor or hinder the construction of the professional role from the initial stages of training.

### Objective

The study aims to explore the perceptions, emotions, and learning experiences of first-year nursing students during their initial clinical simulation encounters with SPs focused on the provision of basic care.

## Methods

### Design

This was a descriptive phenomenological qualitative study aimed at understanding the perceptions of undergraduate nursing students during their first contact with care through simulation experiences with SPs. The phenomenological perspective allows us to perceive the lived human experience and reveal its meaningful understanding as a person [24]. This design sought to explore students’ emotions, expectations, and reactions to their first simulated encounter, as well as their perception of the realism and educational value of the experience. In addition, it allows the identification of learning in communicative, technical, and attitudinal skills, and their impact on confidence and preparation for the real-world clinical environment [25].

### Experience or Role of Researchers

The research team consisted of 5 women and 4 men, including 4 professionals with experience in qualitative research design (EGCB, ATR, PDPH, and MCHC). The data were triangulated by 2 external researchers (RJV and MRG). The positioning of the researchers was established in terms of the theoretical framework, their beliefs, previous experience, and personal motivations for participating in the research. The entire team participated in the evaluation of each stage of the research process to reduce researcher bias.

### Participants and Sampling

This study was conducted at a university in the Community of Madrid, Spain. In this country, the undergraduate degree in nursing consists of 4 academic years. Specifically, the course Introduction to Practice: Basic Care, which is part of the curriculum, is worth 6 European Credit Transfer and Accumulation System credits and is taught in the first year in the second semester. A purposive sampling strategy was applied with the following selection criteria: (1) being first-year students of the nursing degree, (2) enrolled for the first time in the course Introduction to Practice: Basic Care, (3) not repeating or previously enrolled in the module, (4) having participated in at least 1 complete simulation session with a SP (range: 1-4 sessions), and (5) providing informed consent to participate in the study.

It should be noted that the students have not yet had direct contact with the hospital environment, since clinical internships begin in the second year during the first semester. Therefore, this SBE represented their first structured approach to a clinical situation, albeit in a controlled environment. However, throughout the first course, they have carried out practical training activities in basic skills, such as taking vital signs, patient hygiene, mobilization, and administration of fundamental care, in simulation laboratories with mannequins and other technical resources. These activities allowed them to acquire initial knowledge and skills, as well as a certain familiarity with the simulated clinical environment, which facilitated their participation in more complex scenarios with SPs.

### Data Collection

To capture in-depth the complexity of the experiences during clinical simulations with SPs, a qualitative data collection strategy was implemented, based on a phenomenological approach. This strategy integrated multiple complementary techniques to enrich the understanding of the studied phenomenon from different perspectives. Focus groups (FGs) were conducted within the framework of the simulation sessions and were complemented by field notes prepared by the researchers and reflective narratives written by the participants. This methodological triangulation allowed access to a more holistic and nuanced view of the subjective experiences of the students. The FGs facilitated interaction and the contrast of opinions among participants, favoring the emergence of shared meanings. The field notes, together with the individual narratives, provided additional information of an introspective and observational nature, which contributed to deepening the emotional, cognitive, and attitudinal aspects associated with the simulated experience. Data collection was carried out during April and May 2025.

Each FG consisted of 9‐11 participants, led by 2 different researchers who assumed the roles of moderator and observer. The moderator posed questions to which each participant responded by speaking in turn, while the observer focused on identifying key points and taking detailed notes. This separation of roles was maintained in all 6 FGs to ensure neutrality and enrich the data collection process. A topic guide was used, which was focused enough to collect information about the study area yet open enough to stimulate discussion and interaction among participants (Textbox 1). However, data collection in qualitative studies is flexible; therefore, during the FGs, the moderator asked about those areas of interest that participants raised in relation to the research question [26,27]. All FGs were audio- and video-recorded with the previous permission of participants. The average duration of each FG was 58 minutes, with 6 FGs being conducted, at which point no new information emerged from the data analysis.

Textbox 1. **Interview guide.**

**Postclinical simulation phase**

- What emotions were predominant when facing a “patient” for the first time in a simulated clinical environment?
- Did it change your perception of what it means to provide care?
- Do you think this experience resembles what you might encounter in real clinical practice?
- Do you consider this activity useful for your learning? In what way?
- What aspects would you like to improve before having contact with real patients?
- What elements contributed to the simulation seeming authentic or not?
- How did the presence of the standardized patient influence your way of communicating or behaving?
- Do you think the actor managed to convey emotions or clinical situations in a realistic way?

As a complement to the FGs, field notes were collected by the researchers during the development of the simulations and in the subsequent moments of reflection. These notes recorded nonverbal observations (such as body language, facial expressions, emotional tensions, and spontaneous attitudes), as well as contextual details of the simulated environment, which enriched the interpretation of the discourses collected in the group sessions. A third source of information was also incorporated through written reflective narratives. On a voluntary basis, the participating students were invited to write a personal reflection in the Moodle [SimCapture] virtual environment, responding to a series of open questions aimed at promoting introspection and critical analysis of their experience: “How did you feel during the simulation? What do you think you learned during this experience? How has this simulation influenced your self-confidence in the face of clinical practice?” A total of 43 participants submitted reflective narratives, each providing 1 written response covering the 3 open questions, totaling approximately 3891 words. These narratives allowed access to subjective aspects such as emotions, insecurities, significant learning, and future expectations, providing a complementary view to the group discourses.

The clinical simulation experiences were designed following the SimZones model [28], progressing from lower-complexity zones to more challenging scenarios [15]. In the lower zones (Zone 1 and 2), the focus was on training specific technical skills in a more controlled environment with lower cognitive load. At this stage, teachers adopted an instructional role, directly guiding participants’ learning.

Subsequently, we progressed to Zone 3, where more realistic elements were introduced, such as environmental noise, greater assistance pressure, and the deliberate incorporation of nontechnical skills, such as teamwork, effective communication, and group decision-making. In this phase, the role of the teacher evolved into that of facilitator of inquiry, promoting critical reflection and autonomous learning through structured debriefing strategies.

Table 1 shows the 4 clinical scenarios developed during the simulation, along with the expected learning outcomes and the skills (technical and nontechnical) trained in each.

**Table 1.** Simulated scenario diagram.

Scenario	Learning outcomes	Skills training
A 32-year-old patient, just admitted to the cardiology ward for study, had syncope while at the gym, was seen in the emergency room, and transferred to the ward.	Accurately identify the patient. Correctly apply hand hygiene. Perform the nursing assessment on admission using functional patterns. Introduce and identify oneself appropriately. Provide clear information and resolve the patient’s doubts. Perform nursing interventions related to the patient’s situation.	Hand hygiene Vital signs Basic ECG^a^ Nursing assessment
A 65-year-old patient with chronic venous insufficiency and an infected ulcer in the right internal malleolus. The wound shows signs of infection, so a dressing and culture are required.	Perform a focused nursing assessment. Identify signs and symptoms of infection. Apply the principles of asepsis and antisepsis in wound care. Perform correct sample collection for culture. Develop effective communication with the patient. Correctly document the procedure in the medical record.	Infected wound management Aseptic and sterile wound care techniques Use of gloves
A 78-year-old patient was admitted to the internal medicine ward for prolonged immobility, presenting with a grade 1 pressure ulcer in the sacral or scapular region. Hygiene of the area and postural changes according to protocol are required.	Acquire practical skills in hygiene and pressure ulcer prevention. Perform skin assessment using the Norton Scale. Identify pressure points or risk of UP^b^. Promote patient safety in performing postural changes. Ensure patient comfort during the procedure.	Patient mobilization Privacy Ulcer prevention Management of grade 1 UP
A 24-year-old patient operated for appendicitis, currently in the postoperative period with severe pain (VAS^c^ scale 7/10), is very distressed.	Develop clinical skills in pain assessment and safe administration of medications. Improve therapeutic communication with the patient. Ensure patient safety in the administration of analgesia.	Medication administration: 5 correct Patient identification Pain assessment and management

a ECG: electrocardiogram.

b UP: pressure ulcer.

c VAS: Visual Analogue Scale.

The SPs, who played a variety of roles, were experienced actors with knowledge of caregiving. They were meticulously scripted and skillfully acted out various scenarios, offering participants authentic and immersive experiences to hone both technical and nontechnical skills.

All clinical simulation sessions followed the best practices described by the International Nursing Association of Clinical Simulation and Learning, which encompass 4 key phases [29]: prebriefing, development of the simulated scenario, debriefing, and final debriefing. These phases were meticulously executed and supervised by 2 university professors with expertise in clinical simulation methodology. In particular, during the prebriefing phase, great emphasis was placed on creating a psychologically safe environment following the guidelines proposed by Rudolph et al [30] In addition, the debriefing phase followed the principles of the good judgment model, facilitating an environment in which participants felt empowered to make mistakes, engage in meaningful discussions, and receive constructive criticism. This approach aims to foster reflective learning experiences in which participants and instructors integrate newly acquired knowledge with existing experience [30,31].

Through the simulated scenarios, students not only developed technical and communication skills but also learned how to effectively identify and determine appropriate interventions. The debriefings facilitated reflective thinking and provided constructive feedback, promoting a deeper understanding of the learning experience [32]. As a result, participants are empowered to analyze their actions, identify areas for improvement, and understand the potential impact of their interventions in real-world situations.

### Data Analysis

Verbatim transcriptions for each of the FGs, researchers’ field notes, and the participants’ contributed reflective narratives (RNs) were made. These data were meticulously stored, managed, classified, and organized using the qualitative data analysis software ATLAS.ti (version 24; Scientific Software Development GmbH) [33]. The collected data were analyzed using inductive thematic analysis [34], following the steps proposed by Braun and Clarke [35]: (1) repeated and thorough reading of transcripts; (2) initial coding of relevant fragments; (3) grouping of codes into emerging themes; (4) review and definition of main themes, categories, and subcategories; and (5) interpretation of results in relation to the theoretical framework and study objectives.

This method was selected for its flexibility and ability to identify and interpret significant patterns in the qualitative data. An inductive approach was adopted, involving the systematic identification of relevant text segments to address the research objectives. From these segments, a rigorous process of coding and categorization was carried out through 3 analytical levels: first, subcategories were identified that reflected specific nuances of the experiences; second, these were grouped into categories or subthemes that represented broader and more recurrent aspects; finally, higher-order emerging themes were defined that synthesized the general meaning of the discourses.

This interpretative process was iterative and dynamic, allowing for a deeper and more contextualized understanding of the perceptions, emotions, and experiences expressed by the participants. Four researchers with consolidated experience in qualitative research independently carried out the entire thematic analysis process, from initial coding to the construction of themes, categories, and subcategories. Subsequently, a debriefing was held in which individual proposals were compared, and a consensus was reached on the final structure of the analysis. In cases of discrepancies, these were resolved through joint deliberation until informed and consensual agreements were reached within the team.

### Rigor and Trustworthiness

The study followed the Consolidated Criteria for Reporting Qualitative Research (COREQ) guidelines [36]. The Guba and Lincoln reliability criteria [37] were applied. Data triangulation was conducted among the researchers involved in the analysis, and the analysis process was reviewed by independent researchers to ensure its credibility. Transcripts were provided to participants with the opportunity to add any relevant information. Importantly, anonymity was preserved during this process, as additions were made using assigned alphanumeric codes, ensuring that individual identities remained confidential. Transferability was ensured by a detailed description of the research setting, participants, context, and methods. Confirmability was achieved by introducing variability in participants’ experiences. Each researcher conducted the reading and analysis independently, contrasting and then agreeing on emerging themes, categories, and subcategories.

### Ethical Considerations

Ethical approval to conduct the research was obtained from the UNIE University Research Ethics Committee (CEID2025_09). All participants provided written informed consent before participating in this study. To ensure anonymity and confidentiality, each participant in the FGs and RNs was assigned a unique code.

## Results

### Participants Characteristics

Of the total number of nursing students who met the criteria, 59 (98.33%) of 60 students participated. The majority of participants were female (50/59, 84.75%), compared to 9/59 (15.25%) who were male. The mean age of the participants was 20.4 years (SD 2.75 or 1.83 years; range 17-28 years). The gender discrepancy among students is mainly because the majority of students enrolled in undergraduate nursing programs at Spanish universities are women. The participants were first-year nursing students enrolled in the course Introduction to Practice: Basic Care.

### Themes

Three thematic blocks were identified along with their respective categories and subcategories (Table 2): (T1) first contact with simulation, (T2) learning and competencies, and (T3) preparation for clinical practice.

**Table 2.** Themes, categories, and subcategories.

Theme and category	Subcategories
T1^a^: first contact with the simulation	
C1: initial emotions	Anxiety and nervousness Fear of error Feeling of tranquility
C^b^2: evolution of emotions	Curiosity and motivation Sense of achievement Confidence
C3: impact of realism	Authenticity Experience as real Standardized patient
T2: learning and competencies	
C1: theoretical-practical integration	Application of knowledge Spontaneous activation Meaningful learning
C2: learning from error	Formative value of error Debriefing Punitive evaluation Psychological safety
C3: skills	Clinical assessment Critical thinking and clinical judgment Teamwork
C4: integral perspective	Integral and humanized perspective Active listening Empathy Trust bonding
T3: preparation for clinical practice	
C1: practical self-confidence	Tools Safety in clinical environments Visualization
C2: professional identity	Awareness of professional role Projection into the future

a T: theme.

b C: category.

#### Theme 1. First Contact With Simulation

##### Initial Emotions

The emotional experience of the students during the clinical simulations was intense and diverse, with initial emotions ranging from nervousness to insecurity, surprise, and empathy. Nervousness was a cross-cutting feeling, manifesting itself especially in the wait before entering the stage.

*You are always nervous to see what you find behind the door.* [FG1-3]

This anxiety was related to both uncertainty and the desire to do well.

*I was nervous and scared, but I knew it was to learn.* [FG4-3]

Another participant in her reflective narratives clearly expressed this combination of excitement and fear.

*Very excited but nervous.* [RN17]

However, not all students experienced the stimulation with the same emotional intensity. Some reported greater tranquility, especially when the simulated situation was familiar to them or they perceived that they had mastered the required care.

*I was calm because it was basic care I had seen in class and I did not feel that I was going on an adventure*. [FG1-4]

Emotions intensified when facing emotionally charged situations, showing feelings of insecurity and helplessness.

*The patient was crying and I did not know what to do*. [FG3-2]

and

*I felt bad because I wanted to help but I did not know how.* [FG3-4]

##### Evolution of Emotions

As the SBE progressed, a process of positive emotional evolution was evident in many participants, characterized by a transition from initial nervousness to a greater sense of calm, control, and security. This change was not immediate, but the result of progressive familiarization with the simulated environment, the debriefing, and their own reflections. At first, facing a clinical situation in front of observers and peers generated high levels of anxiety and emotional tension.

*Very nervous at the beginning and more relaxed at the end*. [FG3-2]

and

*At the beginning I was very nervous, but little by little I controlled my anxiety*. [FG5-3]

However, this initial nervousness diminished as they gained confidence in their skills and became more comfortable with the simulated scenario. The safe environment of the simulation, together with the support of the team and emotional self-regulation strategies, such as conscious breathing or concentration, facilitated this transition.

*Learning to breathe and concentrate helped me not to block*. [FG4-8]

These experiences not only made it possible to overcome emotional blocks but also gave way to a growing sense of efficacy and competence. Participants reported feeling increasingly able to function in clinical settings, which contributed to a significant gain in self-confidence.

*At first I was very nervous, but by the end I felt more comfortable*. [FG5-5]

Progressive emotional adaptation thus became a key process for the development of emotional self-regulation, an essential aspect in nursing practice. Repeated exposure to simulated clinical scenarios allowed students to train their ability to cope with stress and remain calm—fundamental skills in real patient care situations.

*I am gaining confidence, losing fear; it gives me confidence for the start of my internship.* [FG6-8]

This reinforces the idea that such experiences prepare them not only on a technical level, but also emotionally.

##### Impact of Realism

Another key aspect was the emotional impact of the realism of the scenarios, which generated an intense affective response and greater involvement on the part of the students. The authenticity of the simulations (both in the setting and in the clinical elements) led to a feeling of total immersion.

*For a moment I thought I was in the hospital*. [FG1-10]

and

*Seeing the ulcer seemed real, unbelievable*. [FG2-11]

This realism generated a deep emotional response, which contributed to the real experience of the simulation.

*It helps me to see myself with that tension inside, with the emotion that I would have in practice*. [FG2-1]

This immersion was especially reinforced by the presence of the SP, whose performance generated a meaningful connection between the student and the simulated situation. Many noted that interacting with an actor, rather than an inert mannequin or simulator, facilitated emotional involvement and the development of more realistic relational skills.

*I like talking to patients, so having it be an actor helps me get into the role*. [FG1-8]

and

*I have learned that practicing in an environment that simulates a nursing room allows me to better lock in knowledge. By performing the techniques step by step, following an order, I feel that they stick with me in a more lasting way*. [RN4]

They expressed that this experience made them feel as if they were really attending to a person in a real health care environment.

*I felt as if I were in the hospital, as if it were a real day.* [FG6-4]

*It was like a real patient... it was very real*. [FG6-10]

In some cases, the actors were able to convey states of vulnerability and anguish that deeply impacted the students, activating empathic responses and emotional containment.

*It seemed very real to me, the patient was crying and she was able to convey those emotions*. [FF3-1]

### Theme 2. Learning and Competencies

#### Theoretical-Practical Integration

Clinical simulation emerged as a fundamental tool to support integration between theoretical knowledge and professional practice in training. The students emphasized that, through experiences in simulated clinical scenarios, they were able to understand in greater depth concepts previously learned in an abstract way.

*I have used the knowledge of what I have been learning, and in the simulation, the things we have learned are clearer.* [RN22]

This process facilitated a resignification of the academic content, as they were able to experience how theory was translated into concrete actions in clinical contexts closely resembling the reality of health care.

*You give the theory and until here you do not see it*. [FG1-6]

Likewise, there was evidence of spontaneous activation of clinical reasoning during practice, where the knowledge acquired was recovered according to situational demands.

*I remembered what we saw in class*. [FG6-2]

The practical experience also supported the organization of previous learning, allowing students to connect content from different participants that are usually presented in a fragmented way in the curriculum.

*Here I feel that I put it in value, that I see it in the patient, in an almost real case*. [FG6-6]

This connection between theory and practice was also manifested in the spontaneous transfer of knowledge during the clinical performance.

*At that moment I thought about what gloves to put on and suddenly I remembered what we saw in class.* [FG6-2]

or

*When I go in there I get the idea that I have to put the thermometer on him because maybe he has a fever because of the infected wound... I start to relate, incredible.* [FG3-10]

This process was also consolidated during the debriefing sessions, in which students collectively reflected on their actions, strengthening the link between theoretical concepts and their clinical application.

*Everything studied makes sense.* [FG5-3]

and

*I have learned that from theory to practice there is a lot of information and you learn a lot.* [RN10]

#### Learning From Error

Another central theme identified in the narratives and the FGs was the formative value of error in the simulated context. The possibility of making mistakes without generating real negative consequences allowed participants to rehearse procedures, recognize failures, and correct them immediately, which was perceived as a unique opportunity for learning.

*I prefer to make mistakes here than in the hospital.* [FG6-10]

This protected experience facilitated the acquisition of skills from psychological safety, minimizing the fear of judgment and stimulating self-reflection. Learning did not occur only from one’s own mistakes, but also through the observation of peers.

*The mistakes of my peers and how to act in similar situations*. [RN27]

The students pointed out that witnessing the actions of others, together with the spaces for shared analysis, allowed them to identify successes and failures that they then incorporated into their own practices.

*I have also learned from the mistakes I have seen or we have talked about, how to heal a wound by watching a patient talking to me.* [FG2-6]

Likewise, the relevance of recognizing one’s own limits and asking for help when necessary was emphasized, an attitude that promotes safety and prevents errors in real clinical practice.

*If you don’t know how to do something, tell someone else or the teacher.* [FG3-11]

and

*Learning to ask for help is part of being responsible with the patient*. [FG4-7]

In FG6, a student reflected this attitude when she said

*I did not know what to take, whether alcoholic or aqueous chlorhexidine, and I said I will ask my partner.* [FG6-5]

The simulation was valued by the students as a safe space for learning, free from the pressure of punitive evaluation.

*I feel at ease that the simulation does not have a grade, that it is formative, that I am not evaluated.* [FG2-8]

This safe environment allowed students to experiment, make mistakes, and learn without fear.

*Nothing happens if I make a mistake, that is good for me because it does not happen to me in clinical practice with a real patient*. [FG2-6]

In this sense, being able to fail without real consequences was fundamental for the learning process.

*Being able to fail and not be penalized is a great advantage for learning*. [FG4-9]

#### Skills

The skills developed through simulation were multiple and essential for professional performance in real environments. First, students highlighted the organization of the clinical assessment process as a key competency. They recognized that properly structuring the collection of information allowed them to act with greater confidence and avoid omissions.

*Knowing how to structure the assessment is very important so that I don’t forget anything*. [FG3-9]

They indicated that this skill can be worked on and perfected through simulation.

*Now I know that I have to follow an order so that I don’t miss anything.* [FG3-10]

and

*It has helped me to create a mental structure, to keep an order, what is important in day-to-day care*. [FG6-7]

However, examples also emerged where the absence of this organization affected performance.

*I was focused on what I had to do. I did not see anything in front of me. I forgot to value the scale and it was in front of me.* [FG6-6]

The development of critical thinking and clinical judgment was another aspect that emerged during the simulations. Students highlighted the usefulness of these scenarios for learning to prioritize interventions, react quickly, and make decisions under pressure.

*I learned to react quickly, to prioritize what is most important*. [FG4-5]

The simulation helped me to think quickly and not get carried away by nerves. [FG5-2]

This type of reasoning was enhanced through experiences close to reality, which allowed the integration of multiple clinical variables.

*It helps to prioritize care, to see the patient as a whole, not in parts*. [FG6-5]

In addition, some indicated that the practice helped them not only to consolidate previous knowledge, but also to generate confidence to incorporate new elements of care.

*To reinforce knowledge, to have more confidence to add new ones.* [FG6-11]

Teamwork was valued as essential to deal with the simulation effectively. Students reflected on the importance of sharing responsibilities, communicating, and coordinating adequately.

*Working as a team helped me feel that I was not alone and that we could solve it together.* [FG4-5]

and

*Thanks to talking with my classmates, it helped me see how important it is to work as a team.* [RN19]

This collaboration also offered emotional security.

*We understood each other just by looking at each other, and that made me feel safe.* [FG6-3]

and

*I did not know what to do, but seeing my classmates act helped me to follow them*. [FG6-2]

However, difficulties in coordination and role definition were also identified, especially at the beginning of the simulation.

*We organized ourselves before going in, but then when we started. we got lost*. [FG1-8]

The lack of clarity generated confusion and loss of time.

*We were all talking at the same time and it was chaos.* [FG6-1]

and

*We had a hard time deciding who did what.* [FG6-8]

These experiences reflect the need to strengthen internal planning and communication to improve team effectiveness in simulated clinical situations.

On the other hand, they especially valued the possibility of interacting with actors, as this allowed them to generate more fluid and credible dialogs. This interaction was key to practicing not only verbal content but also tone, body language, and emotional containment of the patient.

*It helps me to be able to talk to the patient, I think it makes me not feel ridiculous with a simulator.* [FG1-8]

and

*There is a lot of difference from doing the practices with an actor that you can ask*. [FG2-2]

#### Integral Perspective

Participants agreed that participation in these low-simulation scenarios not only allowed them to hone their technical skills but also to incorporate a broader and more humanized view of care. They noted that the simulations helped them to focus on the patient from a holistic perspective, understanding the person beyond the disease. As one student expressed,

*To focus on the individualized patient*. [RN29]

highlighting the change in their approach to more personalized care. Along the same lines, another participant underscored the formative value of these scenarios as a fundamental opportunity to get started in real patient care, allowing them to explore their ability to respond to specific clinical situations.

*From my point of view, I think these simulations are fundamental to be able to start dealing with the patient, to see how you perform and to be able to improve different technical aspects*. [RN14]

The experience also facilitated the development of essential communication skills such as active listening and empathy, key elements in the humanization of care and in building a relationship of trust with the patient. In several FGs, the importance of listening without interrupting and putting oneself in the other person’s place was highlighted.

*Listening to the patient without interrupting helped me to understand his situation*. [FG3-5]

and

*Putting myself in his place made me better understand what he needs.* [FG4-8]

This empathic dimension was also reinforced in FG6, where reflections were shared.

*I think I have learned to listen without interrupting, just letting him tell me how he felt.* [FG6-7]

or

*Feeling what the patient is going through made me care for him in another way, more closely*. [FG6-6]

The simulations also provided a safe environment to rehearse conversations with SPs, which helped improve their communication skills and emotional containment. They were able to practice explaining procedures, adapting to different reactions, and accompanying the patient with serenity.

*She would talk to me about how she felt and I would try to respond calmly, without rushing, as I would in reality.* [FG5-6]

and

*What has helped me most is talking to the patient as if she were on the ward, I would ask her if she was in pain and she would answer me as if she felt it.* [FG6-4]

A particularly valued aspect was the importance of introducing oneself properly and establishing a relationship of trust from the first contact. This initial gesture was interpreted as a concrete expression of nursing professionalism, which directly influences the patient’s perception and well-being.

*How you have to introduce yourself, how to prepare things, has helped me to understand what a nurse’s day-to-day life is like*. [FG2-2]

and

*Introducing yourself with confidence makes the patient relax and trust you more*. [FG4-4]

### Theme 3. Preparation for Clinical Practice

#### Practical Self-Confidence

The SBE not only served to practice technical and communicative skills, but also to help students project themselves into their future role as nurses, consolidate their professional identity, and gain confidence in their own abilities. One of the most valued effects was the possibility of anticipating future clinical experiences, reducing anxiety, and favoring a smoother transition between the classroom and the hospital. The students expressed that, thanks to the simulation, they felt more prepared and less insecure in real situations.

*When I get to practice I will say I have already seen it, I have already felt it again.* [FG1-9]

*The experience has helped me to reflect on how to do better in the hospital.* [FG1-1]

and

*I feel more confident because I have already practiced these situations*. [FG5-4]

This perception was repeated in the individual narratives.

*I think it has helped me a lot because when I go to practice, I will already have had a previous experience, however small it may be*. [RN38]

The students felt that, by facing complex scenarios in a controlled environment, they gained concrete tools to act with greater safety when they have to intervene in a real context.

*I think that if it happens to me in clinical practice, I would know how to react.* [FG6-7]

This is also reflected in the reflective narratives.

*From my point of view, I think that these simulations are fundamental to be able to start dealing with the patient, to see how you manage and to be able to improve different technical aspects*. [RN5]

highlighting the direct usefulness of simulation as preparation for dealing with real situations. This experience also resulted in an increase in self-confidence in the health care environment.

*It helps me to feel confident in a health care context*. [FG6-2]

Along with this sense of familiarity and preparedness, students valued the simulation environment as an opportunity to strengthen self-confidence, decrease fear of error, and develop a sense of self-efficacy. Comments such as

*After the simulation I feel more confident and eager to continue learning*. [FG5-4]

and

*At the end I felt I had done well, and that motivated me a lot*. [FG5-7]

illustrate the positive impact on their perception of competence. Significantly, some recounted how this experience contributed to progressive growth, which directly impacts their peace of mind and emotional performance.

*It has given me self-confidence, and I know that I am capable of solving future situations*. [FG6-7]

The simulation allowed students to identify their emotions, learn to manage them, and experience how they reacted under pressure—an essential aspect of facing the real practice with confidence.

*I realized that I get nervous, but I can control it if I breathe and concentrate.* [FG5-3]

and

*Learning to manage anxiety here helps me for when I have to really take care.* [FG4-8]

This emotional skill was also highlighted in FG6.

*I was very nervous, but seeing that I can stay calm gave me a lot of confidence.* [FG6-2]

and

*Here I could see how I react under pressure, and that helps me to prepare myself.* [FG6-8]

On the other hand, thanks to the SBE, many students visualized themselves more realistically in the immediate future in the clinical setting

*I started by telling her who I was, that I was going to treat her wound...it came out by itself, as if I were already a nurse.* [FG6-5]

This training offered them a kind of rehearsal that facilitated the transition between the academic and the professional role, favoring a clearer mental representation of what they would experience in their first internships. As one participant expressed,

*I see myself as if I were in an internship day*. [RN22]

reflecting how the simulation functioned as a direct projection to the hospital setting.

#### Professional Identity

The simulation not only facilitated technical learning but also actively contributed to the development of professional identity by allowing students to project themselves as nurses in realistic clinical contexts. This experience had a transforming effect on their self-perception, helping them to visualize themselves practicing the profession.

*It made me want to see myself as a nurse*. [FG2-1]

Several participants expressed a strengthening of their professional vocation after the experience.

*It reaffirms that I have chosen the career I like*. [FG2-8]

and

*It has helped me to know that I want to dedicate myself to this.* [RN39]

The possibility of experiencing the role in a simulated way allowed them to become more aware of what professional practice entails.

*When I do the simulation, I see myself in the hospital, really acting as a nurse.* [FG6-10]

and

*I see myself more as a nurse every time I participate.* [FG5-10]

Likewise, students recognized that these practices provided them with a clearer vision of the day-to-day work

*It is a plus to understand what a nurse does on any given day*. [FG1-5]

*Having the opportunity to simulate with real patients allows us to see what a nurse does every day.* [FG3-5]

This perception of anticipated professionalization was also emphasized.

*Here I feel that I am already practicing, even if it is in an internship.* [RN31]

The lived experience also allowed participants to project themselves into the future with greater security and confidence in their abilities.

*To manage future experiences by having the necessary resources and my knowledge.* [RN16]

**Figure 1.**
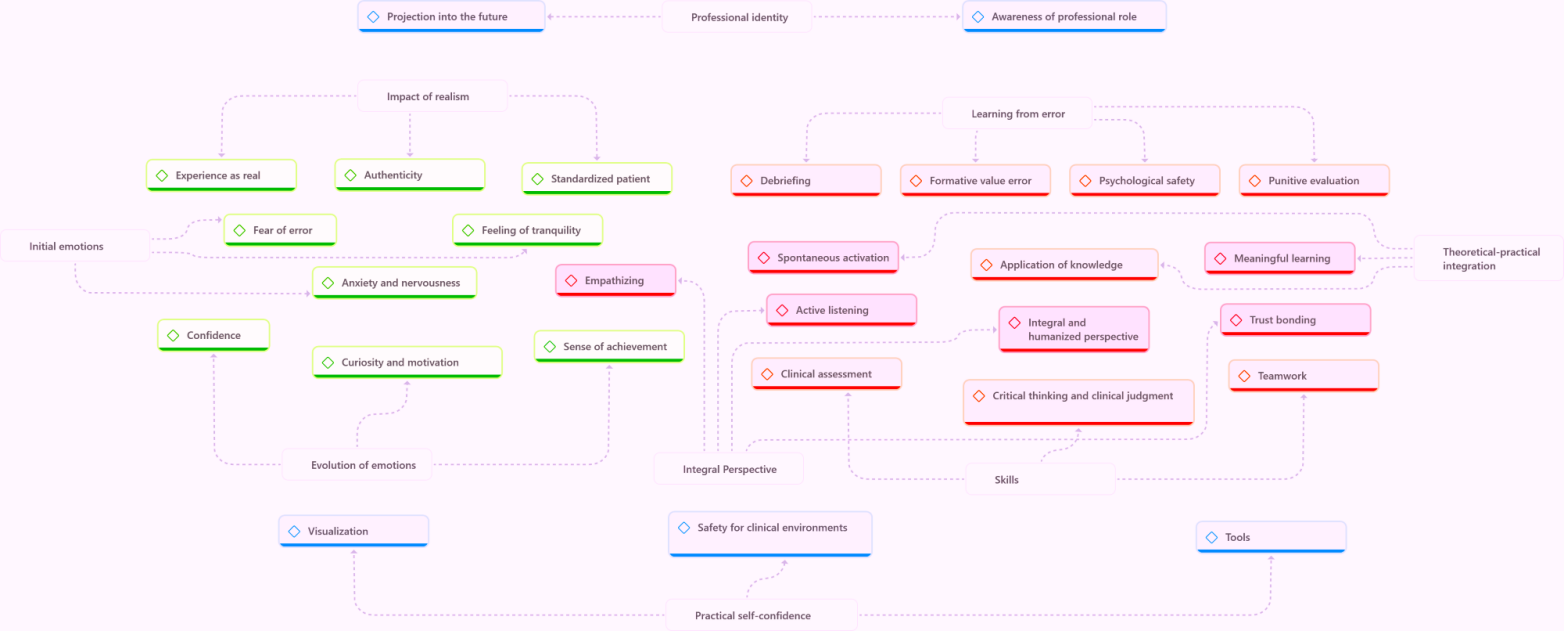
Qualitative data analysis.

Overall, the simulation was experienced as a bridge between the academic and clinical roles, strengthening vocational commitment, identification with the profession, and motivation to continue training as future nursing professionals (refer to Figure 1).

## Discussion

### Principal Findings

The aim of this study was to explore the experiences of nursing students in clinical simulation scenarios focused on the provision of basic care and how these experiences influence their formative, emotional, and professional processes. Clinical simulation, as an active methodology, has proven to be an effective pedagogical tool in the training of health care professionals by providing safe environments where learning is promoted through practice, reflection, and interaction with others [38,39].

Previous studies have supported its usefulness not only in the training of technical skills [5,40], but also in the development of relational and ethical competencies necessary for comprehensive care [17,41,42]. The safe environment provided by the simulation, coupled with the use of strategies such as debriefing and self-regulation techniques, was fundamental in facilitating this emotional transition [43]. These observations are in line with studies [15] that highlight the value of reflective debriefing as a key tool for transforming emotional experience into meaningful learning [44].

From a learning point of view, simulation was consolidated as an effective tool to integrate theory and practice. Students reported a greater understanding of previously abstract concepts and a spontaneous activation of clinical reasoning, as described in research on experiential learning [45,46] and clinical simulation as a catalyst for meaningful learning [47]. Collective reflection during debriefing not only consolidated knowledge but also promoted the critical reconstruction of knowledge from experience [48]. Error, far from being perceived as a failure, emerged as a central formative component. The environment free of real consequences facilitated exploration, self-evaluation, and continuous improvement, confirming previous findings on the value of error in protected contexts. In addition, students highlighted the importance of learning from both their own and their peers’ mistakes, thus strengthening observational learning and collective awareness of clinical performance [30,31]. This reflective and collaborative dimension of the simulation, which includes debriefing as a key moment, reinforced self-confidence, facilitated the sharing of emotions, and supported the building of common meanings about the practice of care. Regarding the competencies developed, the simulation favored the strengthening of essential skills such as structured clinical assessment, critical judgment, decision-making, and care prioritization [49], aspects widely supported in the literature as pillars of nursing education [50]. Likewise, teamwork and interprofessional communication emerged as key components, although challenges related to coordination and role assignment were also evident, underscoring the need for more specific preparation in collaborative competencies [51].

The results are in line with previous research highlighting the value of simulation as an emotionally meaningful experience, capable of arousing empathy, fostering peer collaboration, and strengthening the student’s professional identity [14,52]. In this study, participants highlighted how the realism of the scenarios with SPs, teamwork, and teacher guidance facilitated a deeper understanding of the meaning of caring, beyond the purely technical [16]. The analysis of the verbatim revealed the importance of integrating basic care in clinical simulations, since these not only allow training in fundamental skills, such as hygiene, feeding, or patient comfort, but also constitute a formative space where students explore their emotions, identify their limits, and reflect on their professional role. The process of providing basic care is re-signified by being involved in a simulated context where real people with difficulties, needs, and emotions are represented. This reconstruction of the act of caring allows students to value its relevance and understand it as both a clinical and human act.

In addition, these SBEs become a first approach to the health care environment, especially valuable in the initial courses where students have not yet had access to clinical rotations. Simulation thus acts as a formative bridge that prepares them emotionally and cognitively to face future practices in real contexts with greater confidence and competence [53]. The participants reflected that this experience provides them with security, reduces their fear of the unknown, and strengthens their self-confidence [54] by proving that they are capable of applying their knowledge and managing situations similar to those they will encounter in the clinical setting.

### Practical and Research Implications

This study highlights the value of integrating clinical simulation with SPs from the early stages of nursing education. Practically, the findings support the inclusion of SBEs focused on basic care to facilitate the development of technical, emotional, and communicative skills in a safe and realistic environment. These experiences not only enhance students’ confidence and preparedness for clinical practice but also contribute to the construction of their professional identity.

From a research perspective, the study provides a foundation for further investigations into the emotional and formative impact of early simulation experiences. Future studies could explore longitudinal outcomes, compare different types of simulation modalities, or evaluate the integration of these methodologies across various nursing curricula. In addition, expanding research to diverse educational contexts could enhance the generalizability and applicability of the findings.

### Strengths and Limitations

The strengths of this study lie in its contribution to a deep and experiential understanding of the impact of clinical simulation on the education of nursing students, by exploring not only the development of technical skills, but also emotional, relational, and professional aspects. The research provides an innovative vision of the need to transform traditional teaching models, proposing realistic scenarios that allow for meaningful and reflective learning. In addition, it highlights the usefulness of these simulated experiences to strengthen professional identity and the comprehensive preparation of future nurses.

Among the limitations, it should be noted that the study was carried out with students from a single Spanish university, which restricts the generalization of the results to other educational contexts. In addition, the analysis focused on specific clinical scenarios, so it would be valuable to extend the research with a more diverse sample and with a combination of qualitative and quantitative methods, which would make it possible to evaluate the formative impact of different types of simulation (eg, in the field of mental health). Another limitation is the variability in the number of simulation sessions completed by the participants (ranging from 1 to 4), which may have influenced differences in perceptions and learning experiences. Future studies should control for the number of simulation exposures to analyze its impact more precisely.

Future studies could also consider including second-year students during their first semester of clinical placements. Their participation would allow for valuable comparisons between real clinical practice and simulated practice, further enriching the findings.

### Conclusions

This study shows that clinical simulation has a transformative impact on the education of nursing students by providing a safe, reflective, and emotionally meaningful environment for learning. The emotions experienced, the realism of the scenarios, the interaction with SPs, and the cooperation among peers contribute to the development of comprehensive competencies and the construction of professional identity. Simulation facilitates integration between theory and practice, fosters clinical judgment, decision-making, and allows errors to become a learning opportunity. Debriefing reinforces this process by promoting reflection and consolidation of knowledge from experience. In addition, these experiences act as a first approach to the health care environment, especially useful in the initial stages of the career. Students report an increase in their security and self-confidence, as they see that they can apply their knowledge in simulated clinical contexts. Overall, the results support the need to integrate active methodologies such as simulation into the curriculum, addressing not only technical but also human and emotional aspects of care.

## Supplementary material

10.2196/81617 Checklist 1 COREQ checklist.

## Abbreviations

COREQ: Consolidated Criteria for Reporting Qualitative Research
FG: focus group
RN: reflective narrative
SBE: simulation-based experience
SP: standardized patient
