# Supplementary material for: First Contact With Care Through Clinical Simulation in Nursing Students: Qualitative Study
Source: JMIR Nurs. 2025 Oct 8;8:e81617. doi: 10.2196/81617 (PMC12531447; doi:10.2196/81617)
Supplement: Checklist 1 [file nursing-v8-e81617-s001.pdf]

Consolidated Criteria for Reporting Qualitative Studies (COREQ): 32-item checklist

Developed from:
Tong A, Sainsbury P, Craig J. Consolidated Criteria For Reporting Qualitative Research (COREQ): a 32-item checklist for interviews and focus groups. International Journal for Quality in Health Care. 2007. Volume 19, Number 6: pp. 349 – 357

Item No	Guide Questions/Description	Reported on Page #
**Domain 1: Research team and reflexivity**		
**Personal Characteristics**		
1. Interviewer/ facilitator	Which author/s conducted the interview or focus group?	Pg 3
2. Credentials	What were the researcher's credentials? E.g., PhD, MD	Pg 1
3. Occupation	What was their occupation at the time of the study?	Pg 1,3
4. Gender	Was the researcher male or female?	Pg 1,3
5. Experience and training	What experience or training did the researcher have?	Pg 3,4
**Relationship with participants**		
6. Relationship established	Was a relationship established prior to study commencement?	Pg 4,8
7. Participant knowledge of the interviewer	What did the participants know about the researcher? e.g. personal goals, reasons for doing the research?	Pg 3,4
8. Interviewer characteristics	What characteristics were reported about the interviewer/facilitator? e.g. Bias, assumptions, reasons, and interests in the research topic	Pg 5
**Domain 2: study design**		
**Theoretical framework**		
9. Methodological Orientation and Theory	What methodological orientation was stated to underpin the study? e.g. grounded theory, discourse analysis, ethnography, phenomenology, content analysis	Pg 3,4
**Participant selection**		

Item No	Guide Questions/Description	Reported on Page #
10. Sampling	How were participants selected? e.g., purposive, convenience, consecutive, snowball	Pg 4
11. Method of approach	How were participants approached? e.g., face-to-face, telephone, mail, email	Pg 4,5
12. Sample size	How many participants were in the study?	Pg 4,8
13. Non-participation Setting	How many people refused to participate or dropped out? Reasons?	Pg 4,8
14. Setting of data collection	Where was the data collected? e.g., home, clinic, workplace	Pg 5,6
15. Presence of nonparticipants	Was anyone else present besides the participants and researchers?	
16. Description of sample	What are the important characteristics of the sample? e.g. demographic data, date	Pg 8
**Data collection**		
17. Interview guide	Were questions, prompts, and guides provided by the authors? Was it pilot tested?	Pg 5
18. Repeat interviews	Were repeat interviews carried out? If yes, how many?	
19. Audio/visual recording	Did the research use audio or visual recording to collect the data?	Pg 5
20. Field notes	Were field notes made during and/or after the interview or focus group?	Pg 5,6
21. Duration	What was the duration of the interviews or focus group?	Pg 5,6
22. Data saturation	Was data saturation discussed?	Pg 4,5
23. Transcripts returned	Were transcripts returned to participants for comment and/or correction?	Pg 7,8
**Domain 3: analysis and findings**		
**Data analysis**		
24. Number of data coders	How many data coders coded the data?	Pg 7,8
25. Description of the coding tree	Did the authors provide a description of the coding tree?	Pg 7,8
26. Derivation of themes	Were themes identified in advance or derived from the data?	Pg 9

N
o

Item No	Guide Questions/Description	Reported on Page #
27. Software	What software, if applicable, was used to manage the data?	Pg 8,9
28. Participant checking	Did participants provide feedback on the findings?	Pg 8,9
**Reporting**		
29. Quotations presented	Were participant quotations presented to illustrate the themes/findings? Was each quotation identified? e.g., participant number	Pg 10-16
30. Data and findings consistent	Was there consistency between the data presented and the findings?	Pg 8,9
31. Clarity of major themes	Were major themes clearly presented in the findings?	Pg 10-16
32. Clarity of minor themes	Is there a description of diverse cases or a discussion of minor themes?	Pg 16,17
